# Indolent T-lymphoblastic proliferation with fibrolamellar hepatocellular carcinoma developed after colorectal adenocarcinoma: a case report

**DOI:** 10.3389/pore.2023.1611151

**Published:** 2023-05-12

**Authors:** Wen Han, Bei Wang, Xiang Yong, Yi Zhang, Mingyu Shao, Chun Wang

**Affiliations:** ^1^ Department of Pathology, People’s Hospital of Xinjiang Uygur Autonomous Region, Urumqi, China; ^2^ Graduate Education Department, People’s Hospital of Xinjiang Uygur Autonomous Region, Urumqi, China; ^3^ Department of Pathology, Anhui Wanbei Coal-Electricity Group General Hospital, Suzhou, China

**Keywords:** differential diagnosis, indolent T-lymphoblastic proliferation, iT-LBP, fibrolamellar hepatocellular carcinoma, T-lymphoblastic lymphoma

## Abstract

**Objective:** Indolent T-lymphoblastic proliferation (iT-LBP) is a non-neoplastic disease with an indolent clinical course, manifesting as hyperplasia of immature extrathymic T-lymphoblastic cells. Isolated iT-LBP has been observed, but the majority of iT-LBP cases has been seen in conjunction with other diseases. iT-LBP is easily misdiagnosed as T-lymphoblastic lymphoma/leukemia, and understanding the disease of indolent T-lymphoblastic proliferation may prevent misdiagnosis and missed diagnosis in pathological diagnosis.

**Case presentation:** We report a case morphology, immunophenotypic, and molecular features of iT-LBP combined with fibrolamellar hepatocellular carcinoma developed after colorectal adenocarcinoma and review relevant literature.

**Conclusion:** iT-LBP combined with fibrolamellar hepatocellular carcinoma developed after colorectal adenocarcinoma is relatively rare and should always be considered as a differential diagnosis of T-lymphoblastic lymphoma and scirrhous hepatocellular carcinoma as the two disorders show highly similar clinical features.

## Introduction

The fifth edition of the World Health Organization (WHO) T-cell-dominant tumor-like lesions has recently added indolent T-lymphoblastic proliferation (iT-LBP) [1]. iT-LBP is a newly discovered non-neoplastic disorder characterized by hyperplasia of extrathymic T-lymphoblastic cells and expressing a thymic cortical phenotype. In recent years, reports of iT-LBP have markedly increased. We report a case of iT-LBP combined with fibrolamellar hepatocellular carcinoma developed after colorectal adenocarcinoma and review of related literature.

## Case presentation

### Clinical history

This case involved a 59-year-old male with a liver mass that was observed by physical examination at a local hospital and confirmed by MRI. Follow-up was recommended to the patient. Three months later, MRI re-examination showed that the nodule at the hepatic hilum of the liver square lobe had increased in size. The patient was then referred to our hospital for treatment. The patient had no fever, jaundice, no lymphadenopathy, no history of hepatitis, and had undergone colorectal cancer resection 5 years ago in another hospital. The tumor was very close to the anus and could not preserve the anus and it was performed after a Miles-operation with total rectum extirpation. A pathological report from another hospital showed colorectal adenocarcinoma with pathologic TNM stage T3N0Mx. Abdominal physical examination revealed scars and stoma in the operation area. Blood examination showed normal AFP, whereas creatinine, globulin, LDH, creatine kinase (CK), ALT, and AST increased. Medical ultrasonography (B mode) showed a 34 mm × 32 mm hypoechoic lesion in the left hepatic lobe, with less clear boundary and less uniform internal echo. A cyst in the right kidney was noted; whereas no space-occupying lesions were observed in the left kidney and pancreas. CT showed that the S4 segment of the liver was round in shape, with a slightly low-density shadow with unclear boundary, indicating a need for image enhancement. The mass was surgically removed.

### Macroscopic features

**FIGURE 1:**
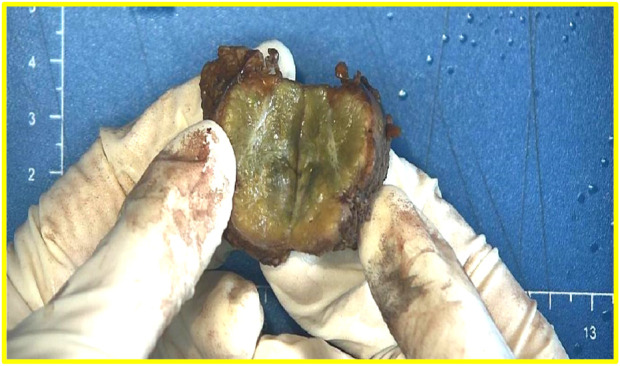
The cut surface of the liver is gray-yellow-grey-green nodules and fibrous scars at the center.

The liver tissue was 4.0 cm × 4.0 cm × 2.5 cm in size and was clinically dissected. Gray-yellow, gray-green nodules were found on the cut surface, with a size of 2.8 cm × 2.7 cm × 2.5 cm, and fibrous scars at the center (Figure 1).

### Microscopic features

**FIGURE 2:**
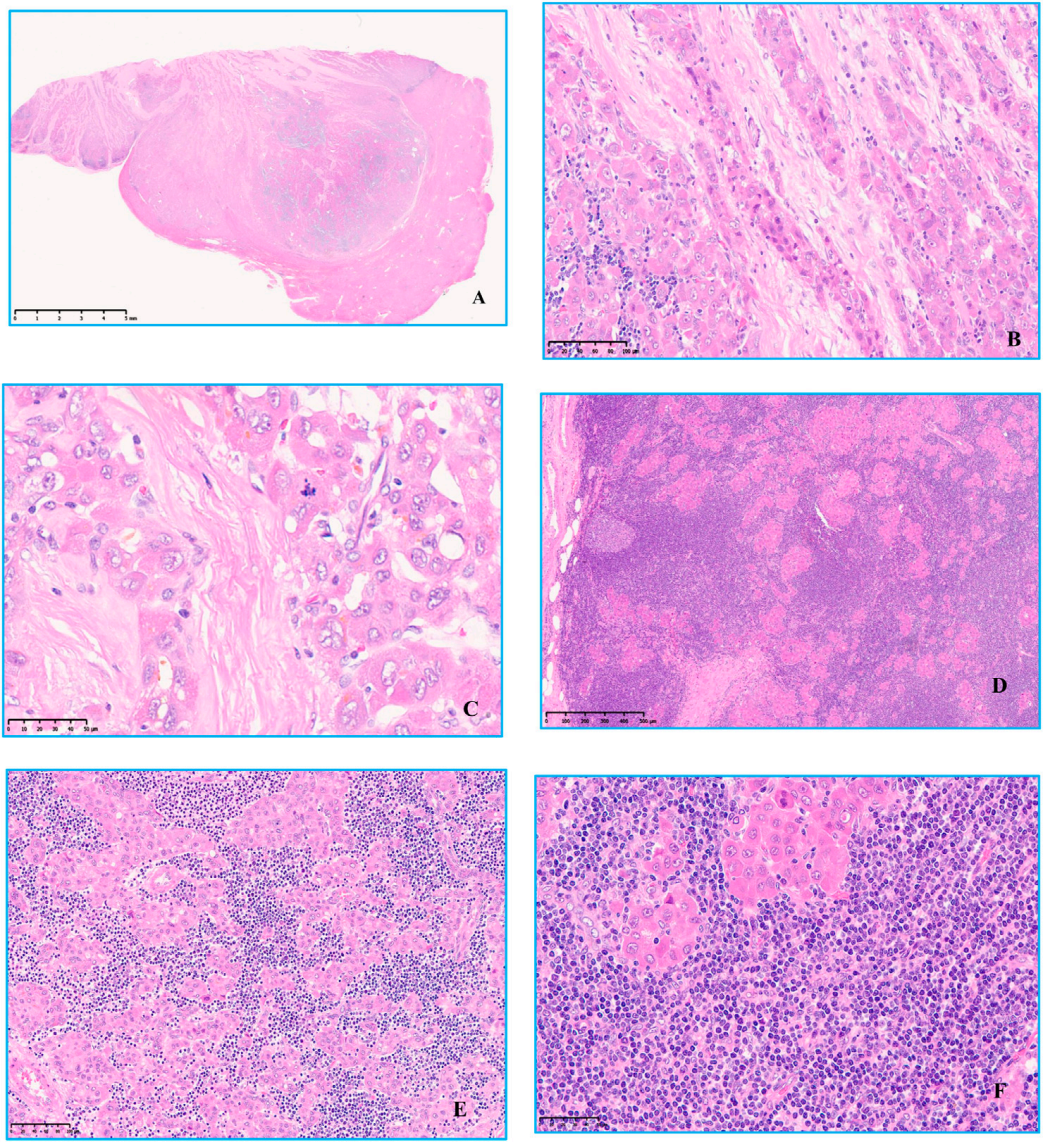
Hematoxylin and eosin staining was observed under microscope. **(A)** Low magnification view Clear boundaries and a thin capsule was observed surrounding the tumor, original magnification ×6. **(B)** Intratumoral fibrous tissue was arranged in parallel, separating tumor cell nests, original magnification ×200. **(C)** The neoplastic hepatocytes have abundant deep eosinophilic cytoplasm, large nuclei, prominent nucleoli, and mitotic figures, original magnification ×400. **(D)** There was a large amount of small lymphocytic cells of uniform shape infiltration between the nests of tumor cells, original magnification ×50. **(E,F)** Some lymphoid cells were small, with few cytoplasm, round nuclei, basophilic nuclei, fine nuclear chromatin, unclear nucleoli, no cell atypia, and no lymphoepithelial lesions, original magnification ×200, original magnification ×400.

A clear border can be seen under low magnification, and a thin capsule was observed surrounding the neoplasm. Intratumoral fibrous tissue was arranged in parallel, separating tumor cell nests. The neoplastic hepatocytes have abundant deep eosinophilic cytoplasm, large nuclei, prominent nucleoli, and mitotic figures. An infiltration of lymphocyte-like cells in clusters or patches could be seen between neoplastic hepatocytes, and these lymphoid cells formed a tumor-like with an area of nodular or sheet-like growth. Secondary lymphoid follicle formation can also be seen. The cells were small to medium-sized, with few cytoplasm, round nuclei, basophilic nuclei, fine nuclear chromatin, indistinct nucleoli, lacked overt atypia, and no lymphoepithelial lesions observed (Figure 2).

### Immunophenotyping

**FIGURE 3:**
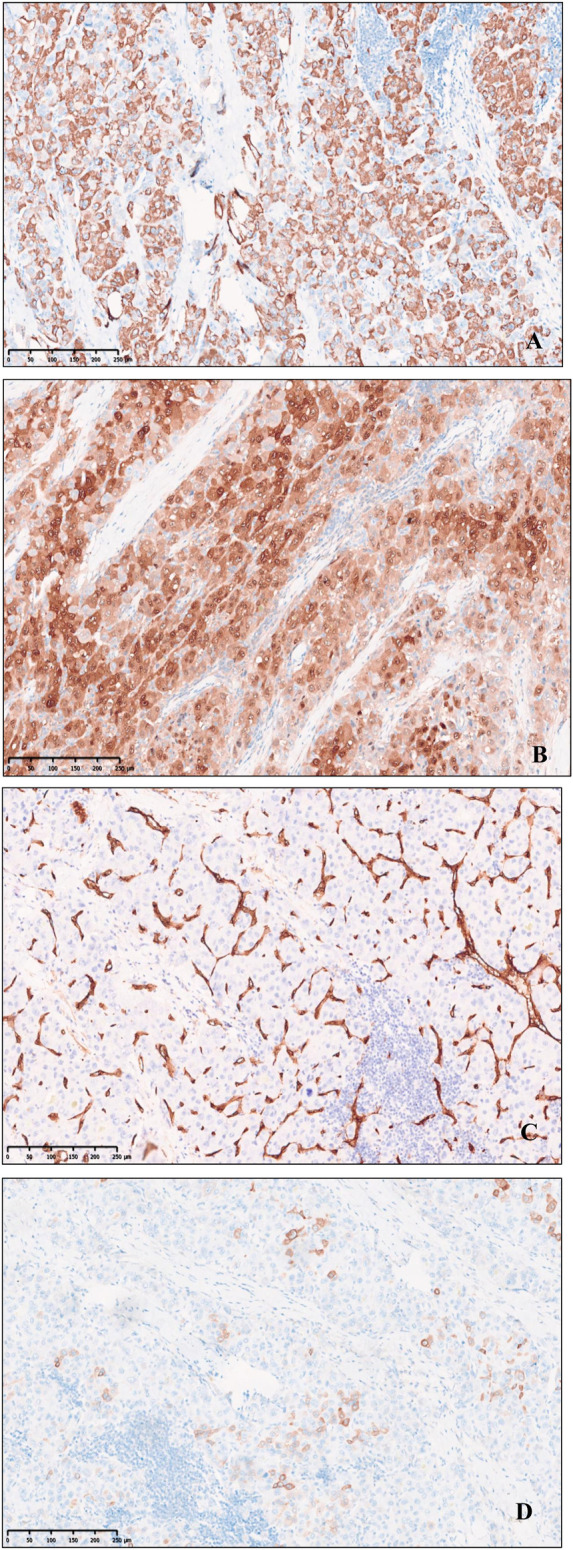
Immunohistochemical expression of Fibrolamellar hepatocellular carcinoma. **(A)** HepPer-1. **(B)** Arginase-1. **(C)** CD34 microvascularization. **(D)** Focal and scattered CK7, original magnification ×100.

**FIGURE 4:**
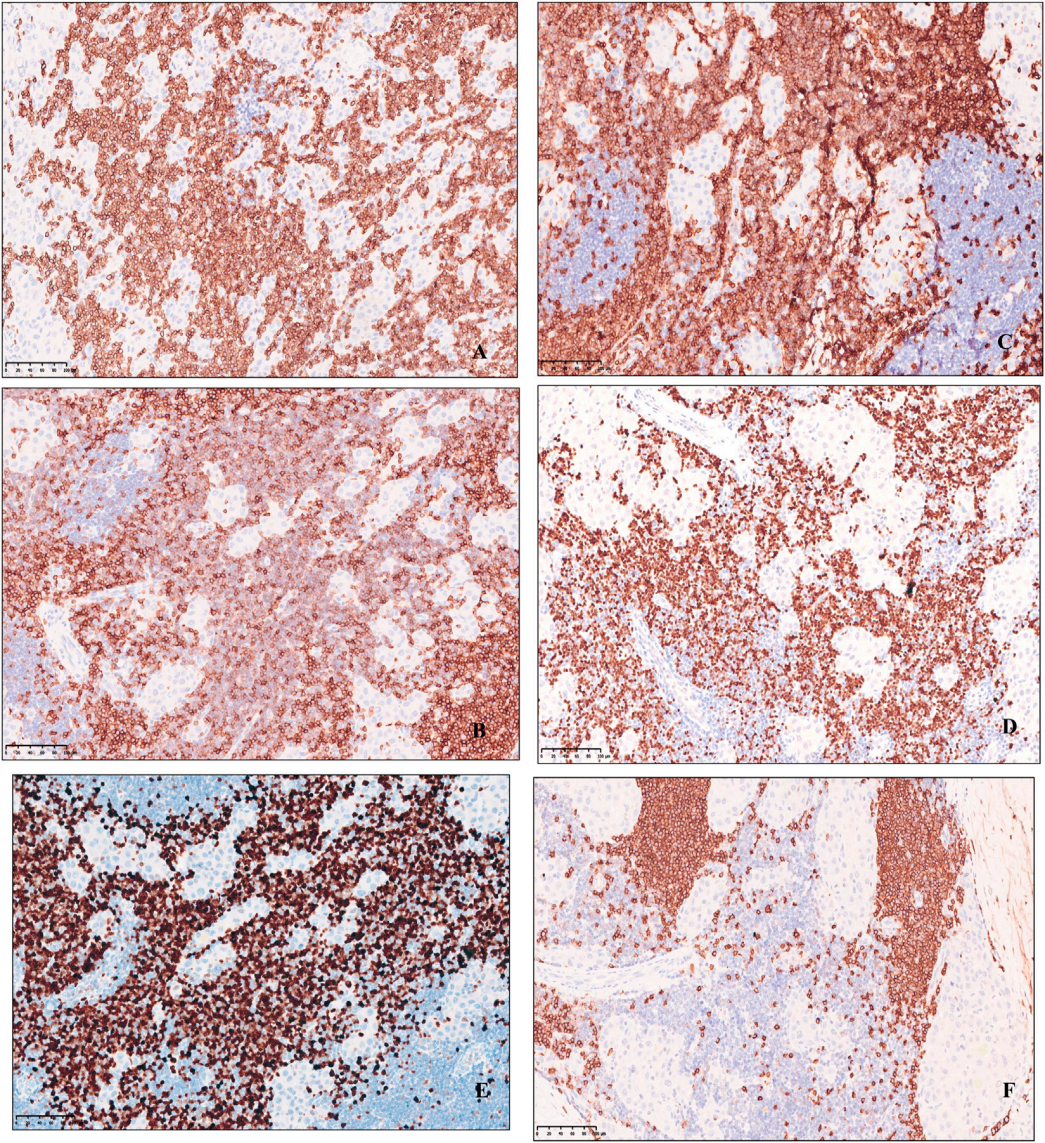
Immunohistochemical expression of small lymphocytic cells of uniform, Lymphoid cells express T cell markers, including **(A)** CD3, **(B)** CD5, **(C)** CD43, **(D)** TDT, **(E)** highly express Ki-67 and **(F)** CD20 negative, original magnification ×200.

Immunohistochemical profile of fibrolamellar hepatocellular carcinoma expresses HepPar1, Glypican-3, Arginase-1, focal or scattered positive for CK7(Figure 3), CD34 is positive in arterialized sinusoids, and there is low ki67 proliferation index, whereas CK19, RB, AFP, β-catenin are not expressed. The proliferative T-lymphoblastic cells to express T cell markers, including CD3, CD5, CD43, as well as TDT, and highly express Ki-67 (Figure 4), but do not express CD34, CD20, MPO, CyclinD1, CD10 and E-cad.

### Molecular analysis

The polymerase chain reaction (PCR) to examine the T-cell antigen receptor gamma and beta gene rearrangement revealed no rearrangement.

## Discussion

Indolent T-lymphoblastic proliferation is a very rare disorder and is characterized by the proliferation of extrathymic T lymphoblasts. In recent years, there has been a relative increase in cases of iT-LBP, perhaps based on our growing understanding of the disease. Isolated iT-LBP has been observed [2], but most of the iT-LBP cases have been seen in conjunction with other disorders, including Castleman disease, hepatocellular carcinoma, follicular dendritic cell tumors, angioimmunoblastic T-cell lymphoma, myasthenia gravis, and acinic cell carcinoma [3–8]. Our patient was diagnosed with iT-LBP with fibrolamellar hepatocellular carcinoma, a special subtype of rare hepatocellular carcinoma that often occurs in young individuals without viral hepatitis or other signs of cirrhosis [9]. AFP levels can be normal or mildly elevated in fibrolamellar carcinoma [10]. Studies have shown that 80% of fibrolamellar carcinoma develops before the age of 35, while only 11% of fibrolamellar carcinoma develops after the age of 40 [11]. This case involved a middle-aged patient with negative AFP expression. Gross tissue analysis showed cicatricial fibrosis at the center of the tumor [12], which is similar to focal nodular hyperplasia. Microscopically, the fibrous tissue in the tumor was arranged in parallel to separate the nest of tumor cells, and the neoplastic hepatocytes have abundant eosinophilic cytoplasm, which may be caused by an increase in the number of mitochondria [13]. The results of immunohistochemical analysis did not support liver metastases from colorectal adenocarcinoma, and thus is described as a primary liver tumor.

We also need to identify hepatocellular adenoma in non-cirrhotic liver tumors, hepatocellular adenoma cut section is soft and uniform, although necrosis, heamorhage, or fibrosis are frequent. The margins of the lesion are ambiguous, grossly and microscopically with little or no fibrous capsule. Microscopically, hepatocellular adenoma is composed of benign hepatocytes arranged in regular plates, usually one or most two cell thick. Tumour hepatocytes have normal cytoplasm, clear cytoplasm, Steatotic cell, or contain pigment. Nuclear and mitoses are rare. Hepatocellular adenoma parenchyma has isolated arteries without bile ducts or multifocal lymphocyte infiltration. TdT-positive lymphocytes are absent in normal liver. The nature of the TdT-positive T lymphocytes infiltrating the tumor in this case is unclear. Given the rarity of this co-occurrence, further studies are needed to determine whether there is a correlation.

There is currently a need to differentiate iT-LBP from T-lymphoblastic lymphoma/leukemia. In this case, the immunophenotype of multifocal clusters of proliferating lymphocytes showed T lymphocytes, and IHC was unable to distinguish T lymphoblastic lymphoma/leukemia. Because the iT-LBP immunophenotype overlaps with T lymphoblastic lymphoma/leukemia expression, and both have high Ki-67 proliferation index expression, we were prompted to consider that this case is T-lymphoblastic lymphoma/leukemia with fibrolamellar hepatocellular carcinoma. However, comparing the two morphologies, T-lymphoblastic lymphoma/leukemia is an aggressive tumor composed of medium-sized blast cells that progress very rapidly when left untreated. In contrast, iT-LBP lymphocytes were small, with little cytoplasm, round basophilic nuclei, fine chromatin, indistinct nucleoli, infrequent mitoses, no cellular atypia, and an indolent clinical course. The patient underwent bone marrow biopsy, with no lymphoma cell infiltration found. The course of disease and the diagnostic criteria proposed by Ohgami et al. [14] can also help us in its diagnosis by distinguishing it from T lymphoblastic lymphoma. In this case, there are many small B lymphocytes surrounding T cells, and lymphoid follicle formation was observed. Reactive hyperplasia and small B cell-derived lymphoma should also be distinguished in differential diagnosis.

All the molecular studies on iT-LBP reported in the literature have confirmed that there is no clonal T-cell receptor (TCR) gene rearrangement [2–7]. Polymerase chain reaction analysis of this patient showed no monoclonal T-cell proliferation. Therefore, T lymphoblastic lymphoma/leukemia was excluded.

To date, Wang [5] and Shin Eun [13] have reported that iT-LBP combined with hepatocellular carcinoma may be sources of immature T cells. Wang suggested that HCC may recruit immature T lymphocytes from the thymic cortex or bone marrow into the tumor by some unknown mechanism. Shin Eun suggested that certain cytokines produced by HCC-transformed hepatocytes may stimulate the proliferation of immature T cells. This clinical feature of our patient may also be due to the abovementioned reasons, although further studies using a larger sample size are necessary to determine the nature and pathogenesis of these immature T cells.

Nine months have passed since surgical resection, but the patient remains well and has shown no tumor recurrence, with CT scans revealing no development of lymphoblastic lymphoma, and peripheral blood tests show no sign of leukemia.

Indolent T-lymphoblastic proliferation combined with hepatocellular carcinoma has been reported in the literature to have a poor prognosis [5, 14]. The prognosis of this case is better than that reported by Wang and Shin Eun, which may be due to the iT-LBP combined with a special type of fibrolamellar hepatocellular carcinoma. However, recent reports suggest that the combination of iT-LPB with hepatocellular carcinoma has nothing to do with the specific type of liver cells [15]. This case is indeed a special type of hepatocellular carcinoma with novel symptoms, further expanding the clinical spectrum of the disease. In conclusion, both iT-LBP and fibrolamellar hepatocellular carcinoma are rare, and their exact pathogenesis and clinicopathological significance have yet to be further elucidated.

## Data Availability

The raw data supporting the conclusion of this article will be made available by the authors, without undue reservation.

## Abbreviations

AFP, alpha-fetoprotein; CT, computed tomography; HCC, hepatocellular carcinoma; iT-LBP, indolent T-lymphoblastic proliferation; LDH, lactate dehydrogenas; MRI, magnetic resonance diffusion tension imaging.
